# Hypoxia-inducible factors in the immunometabolism of metabolic dysfunction-associated steatotic liver disease (MASLD): molecular mechanisms and therapeutic implications

**DOI:** 10.3389/fphys.2026.1779019

**Published:** 2026-02-19

**Authors:** Yinan Zhao, Yige Wang, Faying Li, Guoying Yu

**Affiliations:** 1 Infectious Diseases Major, Qinghai University, Xining, China; 2 Department of Hepatology II, Fourth People’s Hospital of Qinghai Province, Xining, China

**Keywords:** fibrosis, hypoxia, hypoxia-inducible factors, immunometabolism, lipid metabolism, liver inflammation, MASLD, therapeutic targeting

## Abstract

Metabolic dysfunction-associated steatotic liver disease (MASLD) has become a predominant cause of liver disease globally, primarily due to the rising prevalence of metabolic disorders, including obesity and diabetes. The advancement of MASLD from simple steatosis to metabolic dysfunction-associated steatohepatitis (MASH) and fibrosis involves intricate metabolic and immune interactions. Hypoxia-Inducible Factors (HIFs) are integral to the regulation of cellular responses under hypoxic conditions, significantly influencing metabolic homeostasis and modulating immune cell functions. Within the framework of MASLD, HIFs facilitate the adaptive responses to hypoxic conditions and oxidative stress, which are pivotal drivers of disease progression. However, the precise mechanisms by which HIFs influence MASLD pathogenesis remain incompletely understood. This study seeks to investigate the role of HIFs in the immunometabolic processes of MASLD, with particular emphasis on the molecular pathways they regulate within hepatic cells and the immune microenvironment. Furthermore, we examine the challenges associated with therapeutically targeting HIFs, such as the intricate regulation of HIFs, their tissue-specific effects, and the potential risk of inducing tumorigenesis. In conclusion, we underscore prospective research avenues that may yield innovative therapeutic strategies aimed at targeting HIFs to alleviate inflammation, fibrosis, and metabolic dysregulation in MASLD.

## 1 Introduction

Metabolic dysfunction-associated steatotic liver disease (MASLD), previously referred to as nonalcoholic fatty liver disease (NAFLD), has emerged as a significant global health issue, largely attributable to the rising incidence of obesity, diabetes, and metabolic syndrome (Chrysavgis et al., 2022; Zhong et al., 2024). This condition is characterized by the excessive accumulation of fat in the liver, occurring independently of significant alcohol intake, and can progress from simple hepatic steatosis to more severe stages, including metabolic dysfunction-associated steatohepatitis (MASH, previously known as non-alcoholic steatohepatitis or NASH), liver fibrosis, cirrhosis, and ultimately, hepatocellular carcinoma (HCC) (Wang et al., 2025; Powell et al., 2021; Danford et al., 2018). The worldwide prevalence of MASLD has reached concerning proportions, with estimates indicating that it affects approximately 25% of the global population (Powell et al., 2021). In Europe, the prevalence is estimated to range between 20% and 40% (Cianci et al., 2022). With the rise of MASLD and its risk of progressing to a severe liver condition, MASLD is now a key focus in clinical and research areas (Kempiński et al., 2019).

In this context, “Immunometabolism” pertains to the manner in which metabolic alterations, including those mediated by hypoxia-inducible factors (HIF), directly modulate immune cell functions, thereby impacting inflammation and fibrosis in MASLD (Del Río-Moreno et al., 2019). MASLD’s pathogenesis is complex, involving metabolic and immune issues (Shin et al., 2022). Recently, HIFs, which manage cellular responses to low oxygen, have become a focus in liver diseases like MASLD (Holzner and Murray, 2021). These transcription factors control genes related to oxygen balance, metabolism, cell survival, and immunity, activating under hypoxia to help cells adapt (Liu et al., 2020). When the liver is metabolically active and subjected to uneven blood supply or low-oxygen conditions, it frequently undergoes hypoxic stress (Han et al., 2019). As MASLD progresses, hypoxia worsens because of fat buildup, increased metabolic needs, and inflammation (Han et al., 2019; Isaza et al., 2020). HIFs are key in adjusting metabolism, promoting lipid storage, regulating inflammation, and activating hepatic stellate cells, leading to fibrosis (Han et al., 2019; Chen et al., 2019a). They also affect immune cells, driving the chronic inflammation and fibrosis seen in MASH (Wang et al., 2019).

Despite substantial progress in elucidating the role of HIFs in liver pathophysiology, their exact function in the immunometabolic processes of MASLD is not yet fully understood. In particular, further research is needed to clarify how HIFs influence the intricate interplay between metabolic and immune pathways during the progression of MASLD.

Introduce the four main questions that will steer the upcoming sections:

1. How do HIFs contribute to the inflammatory and metabolic changes in MASLD?
2. What is the crosstalk between HIFs and immune cells in the liver in the context of MASLD?
3. How do HIFs regulate lipid metabolism and fibrosis progression in MASLD?
4. What are the potential therapeutic strategies targeting HIFs in the treatment of MASLD?

**FIGURE 1:**
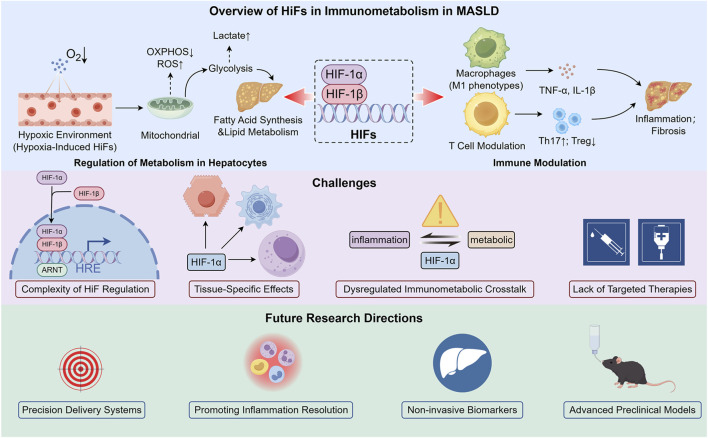
The Role of Hypoxia-Inducible Factors in the Immunometabolism of MASLD. Within the hypoxic microenvironment characteristic of MASLD, HIFs orchestrate crucial metabolic reprogramming. This reprogramming involves redirecting hepatocyte metabolism towards steatosis, while simultaneously facilitating inflammation and fibrosis. Targeting this pathway is challenging due to dysregulated immunometabolic interactions, isoform-specific issues, and major translational hurdles. Future research should focus on creating precise delivery systems and non-invasive biomarkers, validated with advanced preclinical models like humanized mice and liver organoids, to effectively address inflammation and restore metabolic balance.

Figure 1 delineates the multifaceted role of HIFs in the immunometabolic processes of MASLD, highlighting their influence on hepatic metabolism and immune regulation. It also addresses the challenges associated with the therapeutic targeting of HIFs and proposes directions for future research in this evolving field.

## 2 The mechanisms of hypoxia in MASLD and the role of HIFs

Hypoxia is a critical factor in the progression of MASLD, as insufficient oxygen supply in the liver initiates adaptive responses mediated by HIFs (Holzner and Murray, 2021). As MASLD progresses, the accumulation of lipids and the development of insulin resistance intensify hypoxic stress (Holzner and Murray, 2021). HIFs, particularly HIF-1α, modulate metabolic pathways and immune cell functions, thereby promoting inflammation and fibrosis (Liu et al., 2020). This section examines the impact of hypoxia and HIF activation on metabolic and immune processes in MASLD, elucidating their contribution to the disease’s progression from steatosis to more advanced stages, such as MASH and fibrosis.

### 2.1 Mechanisms of hypoxia and activation of HIFs in MASLD

MASLD is closely associated with a disrupted hepatic microenvironment characterized by metabolic disturbances and immune cell infiltration (Zuo et al., 2025). One critical factor contributing to the progression of MASLD is hypoxia, which occurs due to an imbalance between oxygen supply and demand (Han et al., 2019; van der Graaff et al., 2019). As the disease progresses, excessive lipid accumulation in hepatocytes leads to increased oxygen consumption, while the liver’s blood supply becomes insufficient to meet this demand, thereby creating localized hypoxic conditions (Han et al., 2019; van der Graaff et al., 2019; Sundaram et al., 2016).

Hypoxia in the liver activates HIFs, a family of transcription factors that play a central role in the cellular response to low oxygen levels (Tirosh, 2018). HIFs are composed of an oxygen-sensitive α subunit (HIF-1α, HIF-2α, and HIF-3α) and a constitutively expressed β subunit (HIF-1β) (Yan et al., 2024; He Y. et al., 2021). Under normal oxygen conditions, HIF-α subunits are hydroxylated by prolyl hydroxylases (PHDs), leading to their degradation by the von Hippel-Lindau (VHL) complex (Han et al., 2021; Dong et al., 2017). However, in hypoxic conditions, the hydroxylation of HIF-α subunits is inhibited, stabilizing them and allowing them to translocate into the nucleus (Riopel et al., 2020). Once in the nucleus, HIFs bind to hypoxia response elements (HREs) in the promoter regions of target genes, driving the transcription of genes involved in various adaptive responses, including angiogenesis, glycolysis, erythropoiesis, and immune modulation (Tirosh, 2018; Davis et al., 2022; Orlando et al., 2020).

In MASLD, hypoxia predominantly results from the expansion of adipose tissue, the accumulation of lipids within hepatocytes, and heightened metabolic demands, especially in the context of obesity and insulin resistance (Han et al., 2019). The hepatic microvascular alterations observed in MASLD, including endothelial dysfunction and capillary rarefaction, intensify this hypoxic condition, thereby further activating HIFs and their associated downstream signaling pathways (Asai et al., 2017; Vita et al., 2019). HIF-1α is the most extensively investigated isoform in the context of liver diseases, including MASLD, due to its pivotal role in modulating metabolic pathways under hypoxic conditions (He Y. et al., 2021; Luo et al., 2023). In hepatocytes, the activation of HIF-1α facilitates glycolysis and fatty acid synthesis, processes essential for sustaining cellular energy homeostasis during hypoxia (Luo et al., 2023; Lefere et al., 2016). Moreover, HIF-1α upregulates the expression of genes associated with lipid storage, such as those encoding enzymes like fatty acid synthase (FASN) and sterol regulatory element-binding protein 1 (SREBP-1c), which are integral to the pathogenesis of hepatic steatosis (Ezzeddini et al., 2019; Yin G. et al., 2025).

**FIGURE 2:**
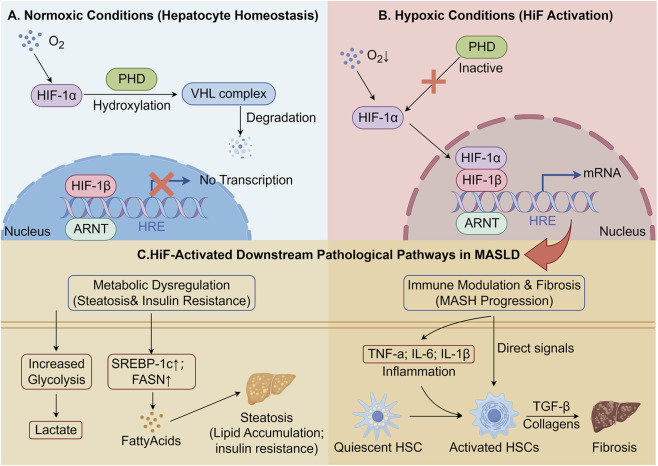
The Mechanisms of HIF Activation driving MASLD Progression. **(A)** Normoxic Conditions: In healthy hepatocytes with adequate oxygen, HIF-α subunits are constantly hydroxylated by PHDs, recognized by VHL, and targeted for proteasomal degradation, preventing transcriptional activity. **(B)** Hypoxic Conditions: Under conditions of hypoxia characteristic of MASLD, PHDs are inhibited. HIF-α stabilizes, translocates to the nucleus, dimerizes with HIF-β, and binds to Hypoxia Response Elements (HREs) to initiate gene transcription. **(C)** Downstream Pathological Pathways: HIF activation drives MASLD progression through two major axes. Metabolically, it upregulates glycolysis and *de novo* lipogenesis genes (SREBP-1c, FASN), leading to steatosis and promoting insulin resistance. Immunologically, it induces pro-inflammatory cytokines (TNF-α, IL-6) which, along with direct signals, activate Hepatic Stellate Cells (HSCs), leading to TGF-β production, collagen deposition, and liver fibrosis.

The role of HIF-1α in hepatic lipid metabolism is also linked to its regulation of inflammation and fibrosis in MASLD (Holzner and Murray, 2021; Wang et al., 2019; Luo et al., 2023). In the hypoxic liver, HIF-1α mediates the activation of hepatic stellate cells (HSCs), which are pivotal in the development of liver fibrosis (Kou et al., 2023; Li et al., 2016). Under hypoxic conditions, HIF-1α upregulates the expression of pro-fibrotic factors such as transforming growth factor-beta (TGF-β) and collagen, leading to ECM deposition and fibrosis progression (Kou et al., 2023; Li Y. et al., 2025; Packer, 2020). In addition to metabolic and fibrotic changes, hypoxia-driven activation of HIFs also impacts immune cell function in the liver (Wang et al., 2019). Hypoxic stress influences the polarization of macrophages, promoting a pro-inflammatory M1 phenotype that exacerbates inflammation in MASLD (Wang et al., 2019; Zhu et al., 2021; Wang et al., 2021). The activation of HIF-1α in macrophages induces the expression of key inflammatory cytokines, including TNF-α, IL-6, and IL-1β, which contribute to the chronic low-grade inflammation observed in MASH and fibrosis (see Figure 2) (Mesarwi et al., 2021).

### 2.2 Metabolic and immune modulation by HIFs in MASLD

In the hypoxic liver, HIFs orchestrate a range of metabolic adaptations to sustain cellular energy homeostasis (Cui et al., 2023). Notably, HIF-1α plays a pivotal role in modulating glucose and lipid metabolism under conditions of reduced oxygen availability (Luo et al., 2023; Yi et al., 2022). Under normoxic conditions, the liver predominantly utilizes oxidative phosphorylation for energy production (Chen et al., 2022; Basheeruddin and Qausain, 2024). However, during hypoxia, HIF-1α activates alternative metabolic pathways that promote anaerobic metabolism (Yi et al., 2022). A critical metabolic adaptation in response to HIF activation is the upregulation of glycolysis (Yi et al., 2022; Xiong et al., 2025). HIF-1α enhances the expression of glycolytic enzymes, such as glucose transporter 1 (GLUT1) and hexokinase, facilitating increased glucose uptake and its conversion to lactate by hepatocytes, even in the absence of adequate oxygen (Xiong et al., 2025). This shift towards glycolysis is particularly pertinent in the context of MASLD, where insulin resistance frequently leads to compromised glucose utilization, thereby exacerbating hepatic steatosis (He Y. et al., 2021). In addition to its role in promoting glycolysis, HIF-1α plays a significant role in lipid metabolism, a critical process in the pathogenesis of hepatic steatosis associated with MASLD (Luo et al., 2023; Cui et al., 2023). HIF-1α upregulates the expression of SREBP-1c, a pivotal transcription factor that governs lipid synthesis and storage (Ezzeddini et al., 2019; Mesquita et al., 2020). Through the activation of genes involved in fatty acid synthesis, such as FASN, HIF-1α facilitates lipid accumulation in hepatocytes, thereby exacerbating MASLD progression (Luo et al., 2023; Ezzeddini et al., 2019). Beyond HIF-1α, accumulating evidence suggests that HIF-2α can also shape hepatic lipid handling and disease progression, and its effects may differ from (or even counterbalance) HIF-1α depending on the cell type and disease context (Mendoza et al., 2023; Hsu et al., 2020; Yang R. et al., 2025).

HIF-1α and HIF-2α are activated by hypoxic stress but they often drive distinct, and sometimes divergent, gene programs in the liver. In hepatocytes, HIF-2α has been repeatedly linked to lipid accumulation and inflammatory injury. Genetic investigations of Vhl-deficient hepatocytes suggest that steatosis, inflammation, and fibrotic remodeling are predominantly dependent on HIF-2α. Furthermore, the targeted deletion of HIF-2α in hepatocytes ameliorates fatty liver, parenchymal damage, lobular inflammation, and the advancement of fibrosis in dietary models of MASLD and MASH (Qu et al., 2011). By contrast, hepatocyte HIF-1α signaling is more consistently connected to fibrogenic pathways in MASLD, including mechanisms that favor collagen remodeling, while its impact on steatosis can vary across experimental settings (Mesarwi et al., 2016). From a therapeutic standpoint, HIF-2α is presently considered a more “drugged” target due to the clinical availability of small-molecule inhibitors. However, systemic blockade of HIF-2α frequently results in anemia (Dogra and Vaishampayan, 2025). Consequently, to optimize therapeutic efficacy and mitigate potential risks, it may be necessary to employ liver-targeted or cell-selective strategies for both isoforms.

HIFs not only aid metabolism but also affect liver immunity, crucial in MASLD progression (Takikawa et al., 2019). Here, the “immunometabolic” perspective is very helpful. The behavior of immune cells is closely related to how these cells use energy and nutrients, and these metabolic choices can directly affect inflammation and profibrotic functions (Kumar, 2019; Gilgenkrantz et al., 2021). In MASLD, especially MASH, HIF-1α partly drives chronic inflammation by influencing liver immune cells (Kou et al., 2023). Under hypoxia, HIFs trigger immune pathways that worsen inflammation and tissue damage, promoting fibrosis (Kou et al., 2023; Cai et al., 2021). A key effect of HIF-1α is altering macrophage polarization (Qiu et al., 2023). Hypoxia activates NF-κB, leading to pro-inflammatory cytokines like TNF-α, IL-1β, and IL-6, which recruit immune cells such as macrophages, neutrophils, and T cells, intensifying MASH’s inflammatory environment (Shao et al., 2025; Wang et al., 2022). HIF-1α also shifts macrophages from anti-inflammatory (M2) to pro-inflammatory (M1), exacerbating inflammation and fibrosis (Takikawa et al., 2019; Wang et al., 2022). Additionally, HIFs have been shown to directly influence the activation of hepatic stellate cells (HSCs), the key effector cells in liver fibrosis (Kou et al., 2023; Tuffs et al., 2025). Under hypoxic conditions, HIF-1α activates HSCs through the upregulation of TGF-β, a potent pro-fibrotic factor (Xu et al., 2024). The activation of HSCs leads to the secretion of extracellular matrix proteins, including collagen, which results in the accumulation of fibrotic tissue and liver stiffness (Kou et al., 2023; Xu et al., 2024). This process is central to the progression of MASLD into MASH and liver cirrhosis. This elucidates a biologically plausible “feed-forward” mechanism in advanced disease, in which hypoxia-induced HIF pathways promote inflammatory activation, while fibrogenesis further intensifies hypoxic stress.

HIFs’ metabolic and immune effects are interconnected, synergistically advancing MASLD. HIF-induced lipid buildup in hepatocytes releases danger-associated molecular patterns (DAMPs), activating immune cells and worsening metabolic issues, creating a cycle of liver damage and fibrosis (Pereira et al., 2023; Liang et al., 2024). Conversely, immune-derived cytokines and metabolic by-products can further reshape hepatocyte metabolism and HSC activation, strengthening a self-reinforcing loop that promotes progression from steatosis to MASH and fibrosis (Subramanian et al., 2022; Gao et al., 2022). This interplay emphasizes the complexity of MASLD and HIFs’ crucial role as disease modulators. In summary, HIFs exert significant influence over both metabolic and immune processes in the liver. By regulating key pathways involved in glucose and lipid metabolism, HIFs promote steatosis and insulin resistance, while simultaneously driving the inflammatory and fibrotic responses that underlie the progression to MASH and liver fibrosis (Wang et al., 2019; Gaucher et al., 2024). Understanding how HIFs coordinate these two processes is essential for developing targeted therapies aimed at halting the progression of MASLD and improving patient outcomes.

### 2.3 HIF-dependent immunometabolic programming of macrophages, neutrophils, and T cells in MASLD

Building on the well-documented tissue-level associations between hypoxia, HIF activation, inflammation, and fibrosis, this subsection seeks to explore a more focused question: how HIFs modulate the intracellular metabolism of key immune cells in MASLD, and how these metabolic changes impact inflammatory or pro-fibrotic responses. In steatotic and inflamed livers, immune cells often face low oxygen, excess lipids, and many cytokines (Ting, 2024). Under these conditions, HIF pathways can be activated by true hypoxia, and also by inflammation-related signals that mimic hypoxic responses (Ting, 2024). These signals influence which energy pathway immune cells rely on (Suzuki et al., 2016). Glycolysis breaks down glucose quickly and can support rapid inflammatory responses (Li HR. et al., 2025). Oxidative phosphorylation (OXPHOS) uses mitochondria to generate energy more efficiently but usually needs enough oxygen (Li HR. et al., 2025). Fatty acid oxidation (FAO) uses fatty acids as fuel and is often linked with longer-term functions and mitochondrial fitness (Leb et al., 2024).

In MASLD, macrophages consist of resident Kupffer cells and monocyte-derived macrophages, with their functions linked to their metabolic state (Drummer et al., 2023; Geric et al., 2018). Generally, inflammatory macrophages favor glycolysis, whereas restorative ones rely more on mitochondrial metabolism. Although not absolute, this pattern helps organize evidence (Geric et al., 2018). HIF-1α can push macrophages toward glycolysis by increasing the expression of glycolytic genes, including GLUT1, HK2, PFKFB3, and LDHA (Liu C. et al., 2023; Zhang et al., 2023). HIF-1α can also induce PDK1, which reduces pyruvate entry into the TCA cycle and can limit OXPHOS (Zhang et al., 2023). This shift is not only about energy supply. It can also affect cytokine output. For example, succinate accumulation can stabilize HIF-1α and promote IL-1β expression in activated macrophages (She et al., 2018). Pyruvate kinase M2 (PKM2) is another metabolic checkpoint that can support HIF-1α activity and IL-1β induction in inflammatory macrophages (Kang et al., 2025). In MASLD, these macrophage-derived cytokines and chemokines can worsen hepatocyte stress, attract more immune cells, and support stellate cell activation, which together can promote inflammation and fibrosis (Wang et al., 2021). Macrophages in MASLD are diverse, with some states being beneficial (Shi et al., 2025). In MASH models, TREM2-linked lipid-associated macrophages aid tissue remodeling and are crucial for fibrosis resolution during disease regression (Shi et al., 2025). This indicates that macrophage metabolism can promote repair in specific contexts (Shi et al., 2025). Thus, the main concern is whether HIF-driven metabolic changes lead macrophages to cause ongoing injury or support balanced remodeling and resolution, depending on the disease stage.

Neutrophils can accumulate in MASH and contribute to injury through oxidants, proteases, and neutrophil extracellular traps (NETs) (Wu et al., 2020; Xu et al., 2026). Because neutrophils rely heavily on glycolysis, they can remain functional in low-oxygen areas, and hypoxia-related HIF-1α signaling has been shown to prolong neutrophil survival, which may increase their persistence in inflamed liver tissue (Morrison et al., 2023; Arelaki et al., 2022). In MASLD and MASH, reviews summarize growing evidence that NETs can amplify hepatic inflammation and may be linked with fibrotic progression, although the exact pathways differ across models (Xu et al., 2026; Yin J. et al., 2025; Yang X. et al., 2025; Du et al., 2022). T cell activation commonly involves increased glycolysis to support proliferation and cytokine production, and hypoxia can further bias these programs through HIF signaling (Nastasi et al., 2021). HIF-1α has been shown to promote Th17 programs while limiting Treg development, linking oxygen sensing to lineage decisions (Singh et al., 2016). In MASLD, Th17 and IL-17 related pathways have been associated with disease progression and fibrosis (Chackel et al., 2016).

In the context of MASLD, HIF pathways have the capacity to alter immune cell metabolism in macrophages, neutrophils, and T cells. A prevalent consequence of this alteration is an increased dependence on glycolysis and diminished mitochondrial flexibility, which can sustain chronic inflammatory signals and facilitate pro-fibrotic interactions with hepatic stellate cells. Concurrently, immune cell metabolic programs exhibit heterogeneity, with certain metabolic states potentially contributing to tissue repair and the resolution of fibrosis under specific conditions. Consequently, the study of immunometabolism offers a valuable framework for comprehending disease progression and identifying therapeutic targets, while emphasizing the importance of specificity regarding cell type and disease stage.

## 3 Challenges and limitations

Despite the significant progress made in understanding the role of HIFs in MASLD, several challenges and limitations remain in fully elucidating their function and translating this knowledge into therapeutic applications. These challenges are critical for the development of effective treatments and for advancing our understanding of the immunometabolic interplay in MASLD.

One of the key challenges in studying HIFs in MASLD lies in the complexity of HIF regulation. HIFs are regulated by multiple factors, including oxygen levels, but also metabolic signals, inflammatory mediators, and other stress conditions, which complicate their precise role in disease progression (Hsu et al., 2020; Haque and Kousoulas, 2019; Luo et al., 2025). The regulation of HIFs is further complicated by the presence of different isoforms (HIF-1α, HIF-2α, and HIF-3α), each of which may have distinct and sometimes opposing effects depending on the tissue context (Jiang et al., 2017; Zhou et al., 2025). In liver disease, these isoforms could have differential roles in metabolic regulation and immune response, which remains poorly understood (Morello et al., 2018). For instance, while HIF-1α is predominantly involved in regulating glycolysis and lipid metabolism, HIF-2α is linked to more specific tissue repair and regeneration processes, with conflicting outcomes depending on the disease stage (Novianti et al., 2019; Yu et al., 2020). This complexity complicates the development of therapies that target HIF pathways, as interventions may need to be isoform-specific and context-dependent.

Within the hepatic environment, HIFs influence a diverse array of cell types, including hepatocytes, HSCs, and immune cells, each exhibiting distinct responses to hypoxic stress (Wang et al., 2019; Kou et al., 2023). For instance, activation of HIF-1α in hepatocytes is associated with lipid accumulation and metabolic dysfunction, whereas its activation in HSCs is linked to the promotion of fibrosis (Luo et al., 2023). These tissue-specific responses complicate the understanding of how HIFs orchestrate various processes and pose challenges in selectively targeting specific cellular responses without disrupting other essential hepatic functions. Under hypoxic conditions, HIFs control metabolic changes like increased glycolysis and lipid buildup, as well as immune responses like pro-inflammatory cytokine activation and fibrosis pathways (Wang et al., 2019; Luo et al., 2023). The interaction between these processes is not well understood, but their dysregulation, where metabolism and inflammation fuel each other, may worsen disease progression. Clarifying how HIFs coordinate these responses is key to finding treatments for both metabolic and immune aspects of MASLD.

Therapeutic translation for chronic metabolic diseases like MASLD faces safety challenges. HIF pathways, which regulate angiogenesis and cell survival, could promote tumor growth, posing risks for MASLD patients prone to HCC (Chen et al., 2019b). Additionally, HIF’s influence on extra-hepatic metabolism could disrupt lipid and insulin regulation in other tissues (Laitakari et al., 2020; Xie et al., 2017). These concerns highlight the importance of liver-targeted delivery, cell-specific strategies, and thorough safety evaluations for HIF therapies in MASLD.

Perhaps one of the most significant limitations in the study of HIFs in MASLD is the lack of targeted therapies. While HIFs are attractive therapeutic targets, their complex regulation and tissue-specific actions make it challenging to develop drugs that specifically modulate HIF activity without causing undesirable side effects (Gonzalez et al., 2018). Current strategies targeting HIFs, such as HIF stabilizers, are still in the early stages of development, and there is a need for more precise and effective treatments (Laitakari et al., 2020). Moreover, given the multifactorial nature of MASLD, therapies aimed solely at modulating HIFs may not be sufficient, and combination therapies targeting multiple pathways may be required for successful treatment.

## 4 Future directions

As our understanding of the role of HIFs in MASLD deepens, several promising future directions have emerged. These directions focus on enhancing therapeutic efficacy, improving diagnostic approaches, and advancing experimental models to better reflect the complexity of human disease.

Future strategies for targeting HIFs in MASLD focus on precision delivery systems for therapies. The challenge is to target liver cells or specific HIF isoforms without affecting other tissues. Advances in nanotechnology and targeted delivery systems, like nanoparticle carriers or RNA-based therapies, offer solutions (Liu MX. et al., 2023). These systems can deliver HIF modulators directly to liver cells, ensuring precise action and reducing side effects (Liu MX. et al., 2023). They also allow controlled release in response to environmental cues, like hypoxia, enhancing efficacy and safety. Furthermore, chronic inflammation drives MASLD progression, especially in MASH (Cai et al., 2021). Future therapies could focus on resolving inflammation rather than just suppressing it (Xia et al., 2024). HIFs are key in immune regulation, and modulating HIF signaling might help resolve inflammation and prevent fibrosis (Xia et al., 2024).

Identifying non-invasive biomarkers for MASLD progression, especially for fibrosis or MASH, remains challenging (Lefere et al., 2017). Advances in genomics, proteomics, and metabolomics may help find biomarkers linked to HIF activation (Hagemann et al., 2022; He D. et al., 2021). Hypoxia-related biomarkers like lactate or specific cytokines could indicate early disease progression, reducing the need for liver biopsies (Bozyel et al., 2025). Imaging technologies could further aid in monitoring hypoxic liver areas, enabling real-time tracking of disease and treatment effects (A et al., 2021). To advance therapeutic development, more advanced preclinical models that accurately mimic human MASLD are needed. Current rodent models fall short in replicating the complexity of human liver disease, especially regarding immune-metabolic interactions and fibrosis. Developing sophisticated models like humanized mice or 3D liver organoids will enhance understanding of MASLD’s molecular mechanisms and aid in testing new therapies, offering more reliable data for clinical applications.

A significant unresolved issue in the field of MASLD immunometabolism is determining whether HIF-mediated metabolic reprogramming within specific immune cell subsets, such as macrophages and T cells, is essential for the progression from simple steatosis to inflammatory MASH and fibrogenesis, or if it primarily represents a secondary response to tissue stress (Morello et al., 2018; Mooli et al., 2021). To explore this subject comprehensively, it is imperative that optimal models are developed to (Chrysavgis et al., 2022): accurately represent physiological oxygen heterogeneity, including zonation-like gradients and controlled hypoxic conditions (Zhong et al., 2024); incorporate multicellular interactions among hepatocytes, stellate cells, endothelial cells, and well-defined immune populations (Wang et al., 2025); enable longitudinal monitoring of transitions between various disease stages; and provide quantitative evaluations of metabolic activity, such as extracellular acidification rate (ECAR) and oxygen consumption rate (OCR) measurements, isotope tracing, and single-cell or spatial profiling (Pereira et al., 2023; Hoogerland et al., 2022). Furthermore, *in vivo* studies utilizing inducible, cell-specific perturbations of HIF-1α and HIF-2α at different disease stages will be essential (Wang et al., 2019).

## 5 Discussion

The role of HIFs in the pathogenesis of MASLD is complex and multifaceted, influencing both metabolic and immune pathways. HIFs are central to the progression of MASLD, driving metabolic dysregulation, lipid accumulation, insulin resistance, and chronic inflammation. Their ability to modulate hepatic metabolism and immune responses places them at the crossroads of the disease’s pathophysiology, making them attractive targets for therapeutic intervention.

Despite the promising potential of HIF-targeted therapies, significant challenges remain. The complexity of HIF regulation, tissue-specific effects, and the dysregulated crosstalk between metabolic and immune pathways complicate the development of precise and effective treatments. Furthermore, the lack of non-invasive biomarkers for disease progression and the need for more advanced preclinical models hinder progress in translating these findings into clinical practice.

Looking ahead, precision medicine approaches, such as targeted delivery systems and therapies aimed at resolving inflammation, offer hope for more effective treatments. Additionally, advances in non-invasive biomarkers and preclinical models will improve our ability to monitor and treat MASLD more accurately. By overcoming these challenges, we can better understand the intricate role of HIFs in MASLD and develop more personalized, effective therapeutic strategies, ultimately improving patient outcomes.

## Data Availability

The raw data supporting the conclusions of this article will be made available by the authors, without undue reservation.
